# Alexithymia, Emotional Distress, and Perceived Quality of Life in Patients With Hashimoto’s Thyroiditis

**DOI:** 10.3389/fpsyg.2021.667237

**Published:** 2021-05-11

**Authors:** Gabriella Martino, Andrea Caputo, Carmelo M. Vicario, Ulla Feldt-Rasmussen, Torquil Watt, Maria C. Quattropani, Salvatore Benvenga, Roberto Vita

**Affiliations:** ^1^Department of Clinical and Experimental Medicine, University of Messina, Messina, Italy; ^2^Department of Dynamic and Clinical Psychology, and Health Studies, Sapienza University of Rome, Rome, Italy; ^3^Department of Cognitive Sciences, Psychological, Pedagogical and Cultural Studies, University of Messina, Messina, Italy; ^4^Department of Medical Endocrinology, Rigshospitalet, Copenhagen University Hospital, Copenhagen, Denmark; ^5^Department of Clinical Medicine, Faculty of Health and Clinical Sciences, University of Copenhagen, Copenhagen, Denmark; ^6^Master Program on Childhood, Adolescent and Women’s Endocrine Health, University of Messina, Messina, Italy; ^7^Interdepartmental Program of Molecular and Clinical Endocrinology and Women’s Endocrine Health, University Hospital, AOU Policlinico “G. Martino,” Messina, Italy

**Keywords:** alexithymia (TAS-20), clinical psychology and health, depression, anxiety, quality of life, HR-QoL, Hascimoto’s thyroiditis, emotional distress

## Abstract

Emotion-processing impairment represents a risk factor for the development of somatic illness, affecting negatively both health-related quality of life (HRQoL) and disease management in several chronic diseases. The present pilot study aims at (i) investigating the associations between alexithymia and depression, anxiety, and HRQoL in patients with Hashimoto’s thyroiditis (HT); (ii) examining the association between these three psychological conditions together with HRQoL, and thyroid autoantibodies status as well as thyroid echotexture in patients with HT; and (iii) comparing the intensity of all these clinical psychological features in patients with HT versus controls. Twenty-one patients with serologically or ultrasonographically verified HT and 16 controls with non-toxic goiter or postsurgical hypothyroidism were recruited for this study. Serum thyrotropin (TSH) and free thyroxine, as well as thyroid autoantibodies (thyroglobulin antibodies and thyroid peroxidase antibodies), were assayed. Alexithymia, depression, anxiety, and HRQoL were assessed with Toronto Alexithymia Scale; Beck Depression Inventory, second edition; Hamilton Anxiety Rating Scale; and Health Survey Short-Form 36, respectively. A negative relationship between the difficulty to describe feelings and the cognitive component of depression was found (*r* = -0.46, *p* = 0.04). Besides, patients with seronegative HT had lower somatic anxiety than patients with HT who tested positive (*r* = -0.68, *p* = 0.01 and *r* = -0.59, *p* = 0.04, respectively). Besides, no statistically significant difference was found between patients with HT and controls with regard to somatic anxiety. The present study suggests the relevance of alexithymia in patients suffering from HT, which may be intertwined with a possible state of underreported depression that is mainly expressed through physical complaints. Promoting the capability to describe and communicate feelings could contribute to psychological elaboration and coping with the disease and, consequently, to the improvement of self-management and perceived HRQoL.

## Introduction

Increasing interest exists regarding the crucial role of psychological factors predicting somatic diseases and influencing the management of chronic illness (Caputo, 2014; Van Houtum et al., 2015; Catalano et al., 2017, 2018, 2019, 2020; Martino et al., 2018a,b, 2020c,d; Conversano, 2019; Kelly et al., 2019; Merlo, 2019; Lenzo et al., 2020; Conversano and Di Giuseppe, 2021). Indeed, chronic disorders pose relevant challenges to patients with specific regard to compliance and adherence, such as entrusting care relationships, taking medications properly, adjusting to new limitations, and changing lifestyle (Castelnuovo et al., 2015; Tomai et al., 2018; Rosa et al., 2019; Aimé et al., 2020; Gugliandolo et al., 2020). This overall requires a better handling of the psychosocial impact of chronic diseases and a paradigm shift in healthcare provision (Caputo, 2015; Van Houtum et al., 2015; Cicero et al., 2017; Conversano et al., 2020; Martino et al., 2020d). Moreover, depression and anxiety are commonly considered two main psychological consequences of chronic disorders, their progression being favored by worsened mental health and perceived health-related quality of life (HRQoL) (Carr-Hill, 1992; Patron et al., 2017; Martino et al., 2019b; Quattropani et al., 2019; Vita et al., 2020) and thus requiring specific clinical psychological treatments (Tomaro et al., 2017; Gangemi et al., 2018; Vicario et al., 2019). Failures in emotion regulation, such as high use of maladaptive defense mechanisms, may in turn contribute to enhance feelings of powerlessness and distress negatively affecting self-care behaviors (Settineri et al., 2019).

In this regard, the conceptual construct of alexithymia—defined as the inability to identify and describe feelings, accompanied by an externally oriented thinking—is of primary importance (Taylor et al., 1999), because an emotion-processing impairment is acknowledged to be a risk factor for the development of somatic diseases, especially in persons affected by physical symptoms of unknown origin (Lumley et al., 1996, 2007; Willemsen et al., 2008; Castelli et al., 2012; Mazaheri et al., 2012; Craparo et al., 2016; Torrado et al., 2018; Marchi et al., 2019; Martino et al., 2020b,c; Prout et al., 2020). The relevance of alexithymia is also supported by the scientific research about the defensive patterns that may emerge in recognizing and elaborating on their illness when patients are not in tune with their affective experience (Di Giuseppe et al., 2014, 2019; Perry et al., 2015; Tesio et al., 2018; Martino et al., 2020a). Also, negative emotional experiences in patients with metabolic syndrome and cardiovascular risk factors have been demonstrated (Bell et al., 2007; Goldbacher and Matthews, 2007).

Among the most frequent endocrinologic pathologies, it is known that hypothyroidism, which is characterized by failure of the thyroid to produce adequate amounts of thyroid hormones, affects approximately 5% of the adult population (Cooper and Biondi, 2012). Hypothyroidism is associated with psychopathological disturbances, such as depression, anxiety, and even poor HRQoL (Crisanti et al., 2001; Cooper and Biondi, 2012; Vita et al., 2013; Winther et al., 2016). Furthermore, patients with hypothyroidism still report residual complaints after replacement treatment with levothyroxine and restoration of euthyroidism. These residual symptoms, including rapid mood changes, depression (fatigue, tearfulness, disturbed sleep, and loss of appetite), and anxiety (concentration problems, mental alertness, and irritability) (Bauer et al., 2001; Grabe et al., 2005; Bell et al., 2007; Samuels, 2008; Bathla et al., 2016; Rieben et al., 2016), may overlap with typical symptoms of psychosomatic disorders (Moncayo and Moncayo, 2014). As a result, HRQoL can be further reduced (Nexo et al., 2014; Boesen et al., 2018a,b). The relevance of these psychological residual symptoms has been highlighted also in patients with Hashimoto’s thyroiditis (HT) (Watt et al., 2012; Moncayo and Moncayo, 2014; Montagna et al., 2016), which is the most common autoimmune thyroid disease and the most common cause of thyroid failure, with hypothyroidism occurring in approximately half of patients with HT (Benvenga and Trimarchi, 2008). Indeed, a high prevalence of psychopathological disorders has been demonstrated in patients with HT (Broniarczyk-Czarniak, 2017), suggesting the importance of personality features and coping strategies of such patients, who could benefit from clinical psychological counseling and support (Yıldız et al., 2017).

To the best of our knowledge, only three studies focused on the relationship between alexithymia and thyroid diseases, one of them including women in the postpartum setting (Le Donne et al., 2012) and two studies including patients with autoimmune thyroid diseases (Ivanova and Gorobets, 2011; Hasegawa et al., 2019). Particularly, Ivanova and Gorobets (2011) concluded that alexithymia is both a risk factor for HT development and a predictor of HT course.

The present cross-sectional, pilot study aims at (i) investigating the associations between alexithymia and depression, anxiety, and HRQoL in patients with HT; (ii) examining the association between these three psychological conditions and HRQoL, and both thyroid autoantibodies status and thyroid echotexture in patients with HT; and (iii) comparing the intensity of such psychological conditions and HRQoL scores in patients with HT versus controls.

## Materials and Methods

### Participants

Hashimoto’s thyroiditis patients and controls were consecutively enrolled from December 2019 through March 2020 at the Department of Clinical and Experimental Medicine of the University Hospital “G. Martino” of Messina, Italy. HT patients had serological and/or ultrasonographic evidence of autoimmune thyroiditis, the first consisting of positivity for one or both thyroid autoantibodies [thyroglobulin antibodies (TgAb) and thyroid peroxidase antibodies (TPOAb)], whereas the second consisted of ultrasonographic evidence of hypoechoic and inhomogeneous echotexture of the thyroid. All patients with HT were on replacement therapy with levothyroxine. Patients with nodular goiter or with levothyroxine-replaced postsurgical hypothyroidism were enrolled as controls. All patients with nodular goiter were euthyroid (i.e., they took neither levothyroxine nor antithyroid drugs), tested negative for both TgAb and TPOAb, and had normal thyroid echogenicity at ultrasound, whereas all patients who had been thyroidectomized were necessarily on levothyroxine. Patients who were thyroidectomized for thyroid nodules, whose serum TgAb and TPOAb were both negative prior to surgery, and whose thyroid histology demonstrated benignity of the nodule and histological absence of lymphocytic thyroiditis were eligible as controls. Exclusion criteria for both HT patients and controls were age <18 years; neuropsychiatric disturbances according to the *Diagnostic and Statistical Manual of Mental Disorders, Fifth Edition* (*DSM-5*) diagnostic criteria (American Psychiatric Association, 2013); and serum thyrotropin (TSH) ≤0.4 or ≥4.0 mU/L. In this regard, controls with nodular goiter and with serum TSH between 0.4 and 1 mU/L or underwent a ^99m^Tc pertechnetate scan were excluded if hyperfunctioning nodules were confirmed (Haugen et al., 2016). Finally, based on the mentioned exclusion criteria, only 21 patients with HT and 16 age-matched controls were eligible and entered the study. Both HT patients and controls underwent blood sampling a few days prior to the visit to detect serum parameters and clinical psychological evaluation at the visit performed by a researcher in clinical psychology.

### Clinical Data and Biochemical Variables

Blood was drawn in the morning (8–9 AM) after an overnight fasting a few days prior to the visit. Serum TSH, thyroxine (FT4), and thyroid autoantibodies were assayed by an immunometric method in patients with HT and controls and evaluated by a physician. Demographics (gender, age, and education level) and body mass index (BMI) were collected during the visit.

### Psychological Assessment

Psychological assessment was conducted by a researcher in clinical psychology, in a confidential setting, performing a gold-standard clinical psychological interview and a psychodiagnostic examination (Rafanelli et al., 2003; Fava et al., 2012), through the Structured Clinical Interview for *DSM-5*—Research Version (SCID-5, Research Version) (First et al., 2015).

The Italian version of the Toronto Alexithymia Scale (TAS-20) (Bressi et al., 1996) was used to measure alexithymic traits. TAS-20 is a self-administered questionnaire comprising 20 items scored on a five-point Likert scale, with a total score ≥61, 60–52, or ≤52 indicating frank alexithymia, possible alexithymia, or normality, respectively. TAS-20 consists of three subscales addressing three main features of alexithymia (Taylor, 2000; Taylor et al., 2003): (i) the difficulty identifying feelings (DIF) subscale, which measures the difficulty in distinguishing between specific emotions and/or bodily sensations related to emotional arousal and contains seven items; (ii) the difficulty describing feelings (DDF) subscale, which indicates the inability to verbalize perceived emotions and contains five items; (iii) the externally oriented thinking (EOT) subscale, which suggests the tendency to focus attention externally instead of considering interior emotional experience and contains eight items. In the present study, the reliability (Cronbach’s α) was 0.75 for the total score, and 0.69, 0.72, and 0.66 for DIF, DDF, and EOT subscales, respectively (Taylor, 2000; Taylor et al., 2003).

The Beck Depression Inventory, second edition (BDI-II), was administered to measure depressive symptoms. It consists of 21 items scored on a four-point Likert scale from 0 (not present) to 3 (severe) (Beck et al., 1996; Ghisi et al., 2006), allowing the detection of somatic–affective depressive symptoms (e.g., agitation, loss of interest, and loss of energy) and cognitive depressive symptoms (e.g., pessimism, guilty feelings, and self-dislike). Total scores of 0–13, 14–19, 20–28, and 29–63 indicate minimal, mild, moderate, and severe depression, respectively. In the present study, the reliability (Cronbach’s α) of the measure was 0.77 for the total score, and 0.69 and 0.70 for the somatic–affective and the cognitive components, respectively (Beck et al., 1996; Ghisi et al., 2006).

The Hamilton Anxiety Rating Scale (HAM-A) was employed to measure anxiety symptoms. It consists of 14 items scored on a five-point Likert scale from 0 (not present) to 4 (severe) (Hamilton, 1959), allowing the detection of psychological symptoms (e.g., anxious and depressed mood, fears, and tension) and somatic symptoms (e.g., cardiovascular, respiratory, or gastrointestinal symptoms). Total scores of 0–13, 14–17, 18–24, and 25–30 indicate minimal, mild, moderate, and severe anxiety, respectively. In the present study, the reliability (Cronbach’s α) of the measure was 0.69 for the total score, and 0.66 and 0.61 for the psychic and the somatic components, respectively (Hamilton, 1959).

The Italian version of the Health Survey Short-Form 36 (SF-36) (Ware and Sherbourne, 1992; Apolone and Mosconi, 1998) was used to measure patients’ perceived HRQoL. SF-36 is a self-report questionnaire comprising eight domains (perceived mental health, emotional role, social functioning, vitality, general health, bodily pain, physical role, and physical functioning). SF-36 total scores range from 0 to 100 points; the lower the scores, the poorer the perceived HRQoL (score of 0 = maximum disability, score of 100 = no disability). SF-36 evaluates patients’ health status by two synthetic indexes, the physical component summary (PCS) and the mental component summary (MCS), which reflect physical and mental well-being, respectively (Ware and Sherbourne, 1992; Apolone and Mosconi, 1998). PCS and MCS values are generally expressed in *t* scores with a general population mean of 50 and a standard deviation (SD) of 10, with highest values indicating better perceived HRQoL. In the present study, the reliability (Cronbach’s α) of the measure was 0.71 and 0.79 for PCS and MCS, respectively (Ware and Sherbourne, 1992; Apolone and Mosconi, 1998).

### Statistical Analysis

Statistical analysis was performed using IBM SPSS, version 25, for Windows. Thyroid autoantibodies and thyroid echotexture were treated as dichotomic variables (positive/negative or altered/normal, respectively). Categorical variables were analyzed by the χ^2^ test or the Fisher exact test, as appropriate. The independent-samples Mann–Whitney *U* test was conducted to compare patients with HT versus controls. Spearman ρ was run to examine the correlations between TAS-20 scores and BDI-II, HAM-A, SF-36 PCS, and SF-36 MCS scores, and between these scores and thyroid autoantibodies status and thyroid echotexture in patients with HT. As the multiple comparisons that we run could inflate type I error (false positives), we further considered the effect size [and relative confidence intervals (CIs)] of the observed relationships, using the following cutoffs: *r* values of 0.1, 0.3, and 0.5 to test the strength of associations (Cohen, 1988). Finally, *p* < 0.05 was considered significant, whereas *p*-values between 0.05 and 0.10 were considered borderline significant.

### Ethics Statement

The study was approved by the Ethical Committee of the University Hospital “G. Martino,” Messina, Italy, protocol identifying number 80/19, 16/09/2019. This study complies with the 1964 Helsinki Declaration and its later amendments. All participants were adequately informed about the scientific purpose of the study and gave their informed written consent. All data were analyzed anonymously.

## Results

### Clinical Data

Women outnumbered men both in HT patients and in controls with a greater preponderance in the first group [19/21 (90.5%) vs. 10/16 (62.5%), *p* = 0.055]. Age and BMI were similar in the two groups (57.6 ± 13.9 vs. 58.8 ± 10.4 years, *p* = 0.862; and 27.2 ± 5.7 vs. 26.7 ± 4.7 kg/m^2^, *p* = 0.946). Finally, most of participants had secondary or higher education level, with no difference between HT patients and controls [16/21 (76.2%) vs. 13/16 (81.2%), *p* = 0.898].

### Biochemical Variables

Serum TSH and FT4 levels did not differ between patients with HT and controls (1.7 ± 1.0 vs. 1.5 ± 0.8 mU/L, *P* = 0.70; and 15.4 ± 3.9 vs. 15.4 ± 3.9 pmol/L, *P* = 0.96). Of the 21 patients with HT, 14 (66.7%) were positive for both antibodies (TgAb and TPOAb), six patients (28.6%) were negative for both TgAb and TPOAb, and one patient (4.8%) for only TgAb. All the recruited 21 patients had the typical ultrasound features of HT, namely, a hypoechoic and inhomogeneous thyroid parenchyma, except for three patients who were positive for both antibodies.

### Psychological Assessment

Descriptive statistics of the psychological measures for all the 37 participants, as well as for HT and control groups, are shown in Table 1. Regarding alexithymia, 17 patients (46%) scored >60 at TAS-20, thus being alexithymic; 16 patients (43.2%) scored 52–60, thus being possibly alexithymic; and four patients (10.8%) scored <52, thus being non-alexithymic. On average, alexithymia scores were comparable across HT and control groups. All 37 patients (both those with HT and controls) had mild depression (with an overall score between 13 and 19) according to the Italian norms for the BDI-II (Table 1; Ghisi et al., 2006). In contrast, overall anxiety levels were from moderate to severe, as the mean HAM-A score was >17 for HT group and >24 for controls (Table 1). Concerning HRQoL, compared to the Italian norm (50 ± 10) (Apolone and Mosconi, 1998), MCS (33.8 ± 12.4) and PCS (42.4 ± 11.3) scores were generally lower, with a mean difference greater than 1 SD in MCS.

**TABLE 1:** Descriptive statistics (scores) of the psychological measures studied (*N* = 37).

	**HT group (*n* = 21)**		**Control group (*n* = 16)**		**Overall (*N* = 37)**	
**Measure**	**Mean**	**Standard deviation**	**Mean**	**Standard deviation**	**Mean**	**Standard deviation**
TAS-20	59.58	10.33	59.88	6.74	59.70	8.85
TAS-20 (DIF)	17.14	6.18	16.31	5.45	16.78	5.81
TAS-20 (DDF)	14.67	4.82	16.13	4.22	15.30	4.57
TAS-20 (EOT)	27.76	3.45	27.44	3.12	27.62	3.27
BDI-II (total)	17.95	7.54	19.56	9.32	18.65	8.27
BDI-II (somatic–affective)	12.95	5.30	13.69	6.16	13.27	5.62
BDI-II (cognitive)	5.00	3.70	5.87	4.33	5.38	3.95
HAM-A (total)	23.90	7.47	25.00	6.40	24.38	6.95
HAM-A (psychic)	12.43	4.64	13.19	4.04	12.76	4.35
HAM-A (somatic)	11.48	4.30	11.81	3.47	11.62	3.91
PCS	42.90	12.48	41.69	9.78	42.38	11.26
MCS	32.81	12.97	35.06	11.97	33.78	12.43

*TAS-20, Toronto Alexithymia Scale 20-item version; DIF, difficulty identifying feelings; DDF, difficulty describing feelings; EOT, externally oriented thinking; BDI-II, Beck Depression Inventory II version; HAM-A, Hamilton Anxiety Rating Scale; PCS, physical component summary; MCS, mental component summary.*

TAS-20 did not correlate with the other scores in patients with HT, except for DDF, which correlated negatively and moderately with the cognitive component of BDI-II [*r* = -0.46, *p* < 0.05, 95% CI (-0.03, -0.74)] (Table 2).

**TABLE 2:** Correlations between alexithymia and depression, anxiety, and perceived health-related QoL measures in patients with HT (*n* = 21).

		**TAS-20**	**TAS-20 (DIF)**	**TAS-20 (DDF)**	**TAS-20 (EOT)**
BDI-II (total)	Spearman ρ	–0.16	0.22	–0.29	–0.13
	*p*-value	0.480	0.334	0.203	0.567
BDI-II (somatic–affective)	Spearman ρ	–0.10	0.18	–0.07	–0.19
	*p*-value	0.656	0.434	0.768	0.409
BDI-II (cognitive)	Spearman ρ	–0.16	0.18	–0.46	0.04
	*p*-value	0.499	0.440	**0.037**	0.875
HAM-A (total)	Spearman ρ	0.08	0.15	0.05	0.07
	*p*-value	0.728	0.514	0.812	0.762
HAM-A (psychic)	Spearman ρ	0.12	0.23	0.07	0.18
	*p*-value	0.595	0.323	0.777	0.433
HAM-A (somatic)	Spearman ρ	–0.10	–0.06	–0.03	–0.13
	*p*-value	0.660	0.793	0.887	0.584
PCS	Spearman ρ	–0.23	–0.03	–0.35	0.03
	*p*-value	0.313	0.911	0.119	0.892
MCS	Spearman ρ	0.07	–0.28	0.29	0.25
	*p*-value	0.756	0.221	0.203	0.272

*TAS-20, Toronto Alexithymia Scale 20-item version; DIF, difficulty identifying feelings; DDF, difficulty describing feelings; EOT, externally oriented thinking; BDI-II, Beck Depression Inventory II version; HAM-A, Hamilton Anxiety Rating Scale; PCS, physical component summary; MCS, mental component summary. Statistically significant *p*-values (*p* < 0.05 minimum) are indicated by the boldface print.*

In patients with HT, thyroid autoantibodies status was associated with anxiety levels. Indeed, TgAb negativity correlated with lower somatic anxiety levels (*U* = 15.50, *p* = 0.013, *r* = -0.68) (Table 3). As expected, TgAb-positive patients had higher scores of the somatic component of HAM-A compared with TgAb-negative patients (median = 12.5 vs. 7). A negative relationship was also found between TPOAb positivity and somatic anxiety levels (*U* = 18.50, *p* = 0.042, *r* = -0.59) (Table 4), with TPOAb-positive patients having higher somatic HAM-A score than their negative counterparts (median = 12.0 vs. 8.0). Finally, the typical thyroid echotexture of HT was not associated with any of the psychometric tests (Table 5).

**TABLE 3:** Associations between TgAb and psychological measures in patients with HT (*n* = 21).

				**95% CI for rank-biserial correlation**	
	***U***	***p***	**Rank-biserial correlation**	**Lower**	**Upper**
TAS-20	59.50	0.455	0.21	–0.31	0.64
TAS-20 (DIF)	56.00	0.626	0.14	–0.37	0.59
TAS-20 (DDF)	58.50	0.500	0.19	–0.33	0.62
TAS-20 (EOT)	48.00	0.970	–0.02	–0.50	0.47
BDI-II (total)	53.00	0.794	0.08	–0.42	0.55
BDI-II (somatic–affective)	46.50	0.880	–0.05	–0.53	0.45
BDI-II (cognitive)	53.00	0.792	0.08	–0.42	0.55
HAM-A (total)	29.00	0.144	–0.41	–0.75	0.10
HAM-A (psychic)	42.50	0.653	–0.13	–0.58	0.38
HAM-A (somatic)	15.50	**0.013**	–0.68	–0.88	–0.29
PCS	56.00	0.627	0.14	–0.37	0.59
MCS	49.00	1.000	0.00	–0.49	0.49

*TAS-20, Toronto Alexithymia Scale 20-item version; DIF, difficulty identifying feelings; DDF, difficulty describing feelings; EOT, externally oriented thinking; BDI-II, Beck Depression Inventory II version; HAM-A, Hamilton Anxiety Rating Scale; PCS, physical component summary; MCS, mental component summary. Rank-biserial correlation indicates the difference between TgAb negativity and positivity. Statistically significant *p-*values (*p* < 0.05 minimum) are indicated by the boldface print.*

**TABLE 4:** Associations between TPOAb and psychological measures in patients with HT (*n* = 21).

				**95% CI for rank-biserial correlation**	
	***U***	***p***	**Rank-biserial correlation**	**Lower**	**Upper**
TAS-20	63.5	0.160	0.41	–0.12	0.76
TAS-20 (DIF)	58.00	0.329	0.29	–0.26	0.69
TAS-20 (DDF)	64.00	0.148	0.42	–0.11	0.77
TAS-20 (EOT)	37.00	0.555	–0.18	–0.63	0.36
BDI-II (total)	40.50	0.755	–0.10	–0.58	0.43
BDI-II (somatic–affective)	37.00	0.556	–0.18	–0.63	0.36
BDI-II (cognitive)	41.00	0.783	–0.09	–0.57	0.44
HAM-A (total)	32.50	0.348	–0.28	–0.69	0.27
HAM-A (psychic)	45.50	1.000	0.01	–0.50	0.52
HAM-A (somatic)	18.50	**0.042**	–0.59	–0.84	–0.12
PCS	47.00	0.907	0.04	–0.47	0.54
MCS	48.00	0.845	0.07	–0.46	0.56

*TAS-20, Toronto Alexithymia Scale 20-item version; DIF, difficulty identifying feelings; DDF, difficulty describing feelings; EOT, externally oriented thinking; BDI-II, Beck Depression Inventory II version; HAM-A, Hamilton Anxiety Rating Scale; PCS, physical component summary; MCS, mental component summary. Rank-biserial correlation indicates the difference between TPOAb negativity and positivity. Statistically significant *p-*values (*p* < 0.05 minimum) are indicated by the boldface print.*

**TABLE 5:** Associations between the echotexture of the thyroid and psychological measures in patients with HT (*n* = 21).

				**95% CI for rank-biserial correlation**	
	***U***	***p***	**Rank-biserial correlation**	**Lower**	**Upper**
TAS-20	25.50	0.920	–0.06	–0.65	0.58
TAS-20 (DIF)	13.50	0.190	–0.50	–0.85	0.17
TAS-20 (DDF)	32.00	0.650	0.18	–0.49	0.72
TAS-20 (EOT)	40.00	0.204	0.48	–0.19	0.85
BDI-II (total)	9.50	0.087	–0.65	–0.90	–0.05
BDI-II (somatic–affective)	12.50	0.156	–0.54	–0.87	0.12
BDI-II (cognitive)	8.50	0.068	–0.68	–0.92	–0.12
HAM-A (total)	33.00	0.579	0.22	–0.46	0.74
HAM-A (psychic)	23.00	0.724	–0.15	–0.70	0.52
HAM-A (somatic)	44.00	0.096	0.63	0.02	0.90
PCS	25.50	0.920	–0.06	–0.65	0.58
MCS	39.50	0.227	0.46	–0.22	0.84

*TAS-20, Toronto Alexithymia Scale 20-item version; DIF, difficulty identifying feelings; DDF, difficulty describing feelings; EOT, externally oriented thinking; BDI-II, Beck Depression Inventory II version; HAM-A, Hamilton Anxiety Rating Scale; PCS, physical component summary; MCS, mental component summary. Rank-biserial correlation indicates the difference between normal and hypoechoic and inhomogeneous echotexture of the thyroid.*

No significant difference was found in scores obtained from the psychodiagnostic evaluation between patients with HT and controls (Table 6).

**TABLE 6:** Differences between patients with HT and controls concerning the psychological measures (*n* = 37).

				**95% CI for rank-biserial correlation**	
	***U***	***p***	**Rank-biserial correlation**	**Lower**	**Upper**
TAS-20	174.00	0.866	0.04	–0.33	0.39
TAS-20 (DIF)	153.50	0.667	–0.09	–0.44	0.29
TAS-20 (DDF)	202.00	0.303	0.20	–0.17	0.53
TAS-20 (EOT)	165.00	0.939	–0.02	–0.38	0.35
BDI-II (total)	182.00	0.679	0.08	–0.29	0.43
BDI-II (somatic–affective)	185.00	0.611	0.10	–0.27	0.45
BDI-II (cognitive)	184.00	0.633	0.09	–0.28	0.44
HAM-A (total)	183.00	0.656	0.09	–0.28	0.44
HAM-A (psychic)	184.00	0.634	0.09	–0.28	0.44
HAM-A (somatic)	185.50	0.601	0.10	–0.27	0.45
PCS	162.00	0.866	–0.04	–0.39	0.33
MCS	185.00	0.612	0.10	–0.27	0.45

*TAS-20, Toronto Alexithymia Scale 20-item version; DIF, difficulty identifying feelings; DDF, difficulty describing feelings; EOT, externally oriented thinking; BDI-II, Beck Depression Inventory II version; HAM-A, Hamilton Anxiety Rating Scale; PCS, physical component summary; MCS, mental component summary.*

## Discussion

Although autoimmune thyroid diseases are considered risk factors for both depression and anxiety (Bauer et al., 2001; Grabe et al., 2005; Bell et al., 2007; Samuels, 2008; Bathla et al., 2016; Rieben et al., 2016), the relationship between autoimmune and non-autoimmune thyroid diseases and alexithymia has been poorly studied so far (Ivanova and Gorobets, 2011; Le Donne et al., 2012; Hasegawa et al., 2019).

In our study, we found an overall prevalence of frank or possible alexithymia in 89.2% of cases, supporting the hypothesis that patients with HT and controls (patients with thyroid diseases in general) may be affected by difficulty in identifying and describing feelings to a significant extent, thus focusing on external events rather than on inner experiences (Hasegawa et al., 2019). Mean BDI-II, HAM-A, and SF-36 scores revealed mild depression, severe anxiety, and lower mental HRQoL compared to the normative Italian samples. By and large, a significant degree of psychological suffering accompanied by the perception of lacking personal resources to face emotional challenges has emerged in patients with thyroid diseases (Bianchi et al., 2004; Dayan and Panicker, 2013). From such a perspective, the overwhelming emotions related to chronic disease may threaten/affect integration, thus becoming undifferentiated and unmodulated (Luminet et al., 2018). This hypothesis is supported by the negative association between the difficulty in describing feelings and depression, especially the cognitive component, found in HT patients. Therefore, the impaired emotional competence may have a role in elaborating on illness experience and managing thyroid diseases, as also found in previous studies about other chronic conditions, such as type 2 diabetes, inflammatory bowel diseases, and psoriasis (Porcelli et al., 1996; Pollatos et al., 2011; Avci and Kelleci, 2016; Talamonti et al., 2016; Amiri and Behnezhad, 2019; Martino et al., 2019a,c). However, the negative association between the difficulty in describing feelings and depression seems apparently counterintuitive as previous research has demonstrated that higher alexithymia makes patients more vulnerable to experience depression (Hemming et al., 2019). Indeed, as shown in the current study, the less patients with HT are able to express their emotions, the less they experience cognitive depressive symptoms. This association may suggest a possible underreporting of depression (which is mainly expressed through physical complaints) in patients with HT, who could live with unrecognized feelings of pessimism, guilt, or self-dislike, as their levels of depression could be emotionally denied. From a psychodynamic perspective, their difficulty in emotion recognition and processing may be a defensive response aimed at preventing painful experiences of grief and loss, with consequent impaired psychological mourning elaboration (Caputo, 2013, 2019; Shahar and Lerman, 2013; Marchini et al., 2018). Indeed, poor symbolic and emotional capacity has been found to positively correlate with maladaptive and less mature defense mechanisms, which could suppress negative emotions as an unconscious ego’s function to protect the self (Marchini et al., 2020).

Another interesting finding was the positive correlation between somatic anxiety and thyroid autoantibodies status in patients with HT, assessed through TgAb and TPOAb levels that provide serological evidence of autoimmune thyroiditis. This appears in line with a previous study showing that thyroid autoimmunity may be a high risk factor for anxiety disorders, as psychopathological disturbances and the autoimmune reaction are hypothesized to be rooted in the same aberrancy in the immunoendocrine system (Carta et al., 2004). From a psychosomatic perspective, residual symptoms despite replacement therapy with levothyroxine in patients with HT might result from somatization in response to physical and psychological stressors (Mizokami et al., 2004; Moncayo and Moncayo, 2014). Indeed, stress is considered as one of the environmental factors potentially affecting immune system directly and indirectly through the nervous and endocrine systems, respectively. Therefore, anxiety experienced at a somatic level can induce immune modulations, in turn contributing to trigger or worsen autoimmune disease, especially in genetically predisposed individuals (Mizokami et al., 2004; Moncayo and Moncayo, 2014).

Finally, no significant difference was found between patients with HT and controls in all the assessed psychological factors (alexithymia, depression, anxiety, and HRQoL). In this regard, we should hypothesize that the small size of both groups may have prevented to reach statistical significance. Another limitation of the present pilot study is its cross-sectional design, which can limit generalizability of our findings. Consequently, no causal relationships can be inferred about the found association between the participants’ psychological status and thyroid autoantibodies. Finally, the use of self-report measures could represent another limitation, despite the gold-standard clinical psychological interview, which conferred a specific objectivity to the performed surveys.

Future larger, longitudinal studies could provide more robust evidence, which is what we aim at performing in perspective research, considering also variables such as disease duration and thyroid-specific HRQoL questionnaires in order to provide more accurate disease-specific information (Watt et al., 2009, 2014, 2015; Wong et al., 2016, 2018). As well, further empirical investigation is needed linking the construct of alexithymia to the construct of defense mechanisms, given the salience of emotion regulation as underlying both such psychological factors, thus contributing to plan and deliver tailored interventions (Lingiardi et al., 2010) in the context of chronic diseases.

## Conclusion

Our study shows that the majority of patients with thyroid diseases—including both those with HT and controls—are alexithymic or potentially alexithymic and that patients with HT may harbor a possible underreporting of depression. Hence, potential benefits may derive from clinical psychological interventions in these patients. Promoting the capability to describe and communicate feelings could contribute to psychological elaboration and coping with thyroid diseases and, consequently, to the improvement of self-management and perceived HRQoL.

## Data Availability Statement

The raw data supporting the conclusions of this article will be made available by the authors, without undue reservation.

## Ethics Statement

The study was approved by the Ethical Committee of the University Hospital “G. Martino,” Messina, Italy, protocol identifying number 80/19, 16/09/2019. This study complies with the 1964 Helsinki Declaration and its later amendments. All participants were adequately informed about the scientific purpose of the study and gave their informed written consent. All data were analyzed anonymously.

## Author Contributions

GM made significant contributions to the design of the research study, drafting of the manuscript, interpretation of the data, and revision of the manuscript. AC performed the statistical analysis, provided the interpretation of data, and gave significant contribution to the draft part of the manuscript. CV, UF-R, and TW provided substantial contribution in the drafting part of the manuscript and revised it critically. MQ and SB critically revised the manuscript. RV provided significant contribution in participants’ recruitment, drafting part, and revision of the manuscript. All authors gave their final approval of the manuscript to be submitted.

## Conflict of Interest

The authors declare that the research was conducted in the absence of any commercial or financial relationships that could be construed as a potential conflict of interest.
